# Diffuse Large B-Cell Lymphoma Germinal Center B-Cell Subtype of the Thyroid

**DOI:** 10.7759/cureus.18893

**Published:** 2021-10-19

**Authors:** Syed Hamza Bin Waqar, Anosh Aslam Khan, Juan Coca Guzman, Susan RS Gottesman, Isabel McFarlane

**Affiliations:** 1 Internal Medicine, State University of New York Downstate Health Sciences University, New York, USA; 2 Internal Medicine, Dow University of Health Sciences, Karachi, PAK; 3 Pathology, State University of New York Downstate Health Sciences University, New York, USA

**Keywords:** epoch, lymphoma, germinal center, gcb, dlbcl, primary thyroid lymphoma

## Abstract

Non-Hodgkin lymphoma is one of the most common hematological malignancies having both nodal and extranodal sites of involvement. The thyroid gland is one of the rarest primary sites. Most cases of primary thyroid lymphoma are diffuse large B-cell in nature; thus, aggressive and in extreme cases can rapidly lead to airway compromise, especially in patients who have been living with goiter for years. We present one such case of a 64-year-old female who presented with signs of airway compromise, requiring emergent airway intubation and surgical debulking. She was treated with emergent chemotherapy (DA-EPOCH-R regimen), without radiotherapy and this resulted in complete remission.

## Introduction

Primary thyroid lymphoma is rare, making up less than 5% of thyroid cancers, and it comprises less than 2.5% of all lymphomas. Diffuse large B-cell lymphoma (DLBCL) accounts for more than half of the cases [1-4]. We report a case of thyroid lymphoma presenting as acute airway compromise in a 64-year-old female with a past medical history of long-standing hypothyroidism. The patient underwent surgical debulking followed by immediate initiation of DA-EPOCH-R therapy without radiotherapy and experienced complete remission without any permanent airway compromise after completing eight cycles of chemotherapy.

## Case presentation

A 64-year-old female with a known history of hypertension, hyperlipidemia, thyroid goiter, and hypothyroidism presented to the tertiary care facility with progressively worsening dysphagia. Patient had a long standing history of Hashimoto disease and was on daily dose of 125μg of levothyroxine with normal thyroid stimulating hormone (TSH) and free T4 levels. The dysphagia was associated with solid and liquid foods, a choking-like sensation, dysphonia, and unintentional weight loss of 10 pounds. These symptoms had progressed within the week before the presentation. Along with these current complaints, the patient also noticed progressive worsening in goiter size, although it had been stable for four years since diagnosis. On examination, the patient was afebrile, tachypneic with a respiratory rate of 27 per minute, and tachycardiac with a heart rate of 116 per minute. She had an elevated blood pressure of 161/97 mm Hg. The patient appeared tired and in no apparent distress; however, she had audible stridor and a raspy voice. Neck exam revealed a large anterior indurated neck mass with flexible fiberoptic nasolaryngoscopy showing clear passage but abutment of supraglottic structures with deviation to the right. The patient was given racemic epinephrine with heliox, which resulted in some comfort, and underwent nasotracheal intubation. Later, she had debulking surgery with gross removal of thyroid tissue to reverse airway compromise. Thereafter, the specimens were sent for pathological analysis.

**Figure 1:**
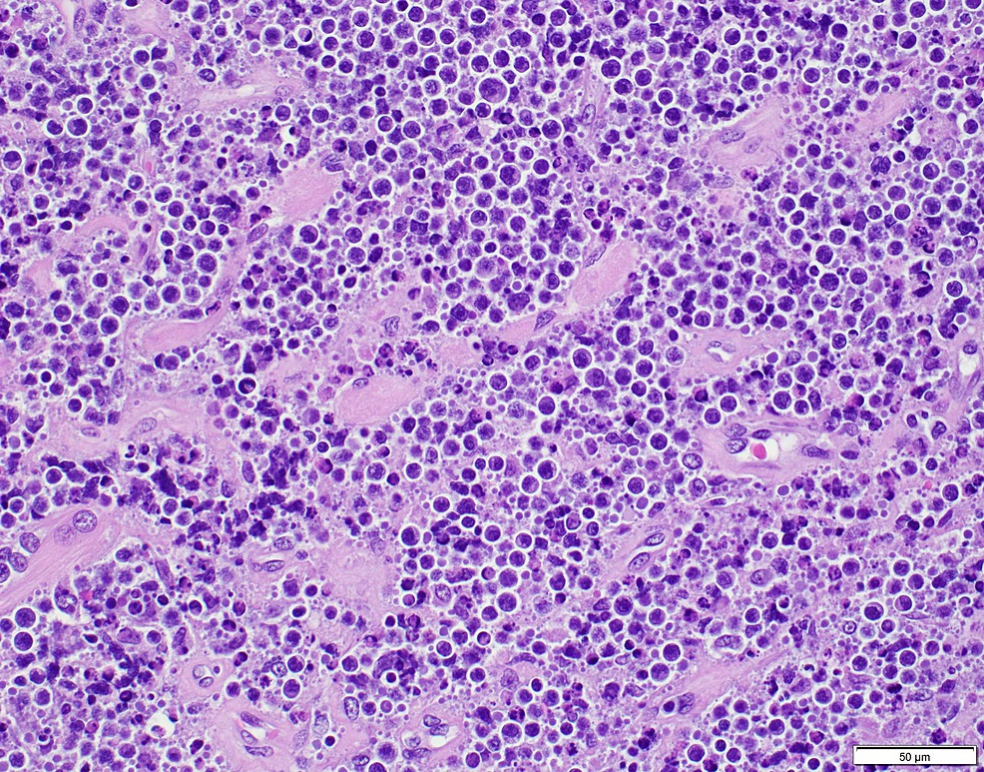
H&E stain at 40x. Diffuse infiltrate of large lymphocytes with prominent nucleoli, a brisk mitotic rate and numerous apoptotic bodies.

**Figure 2:**
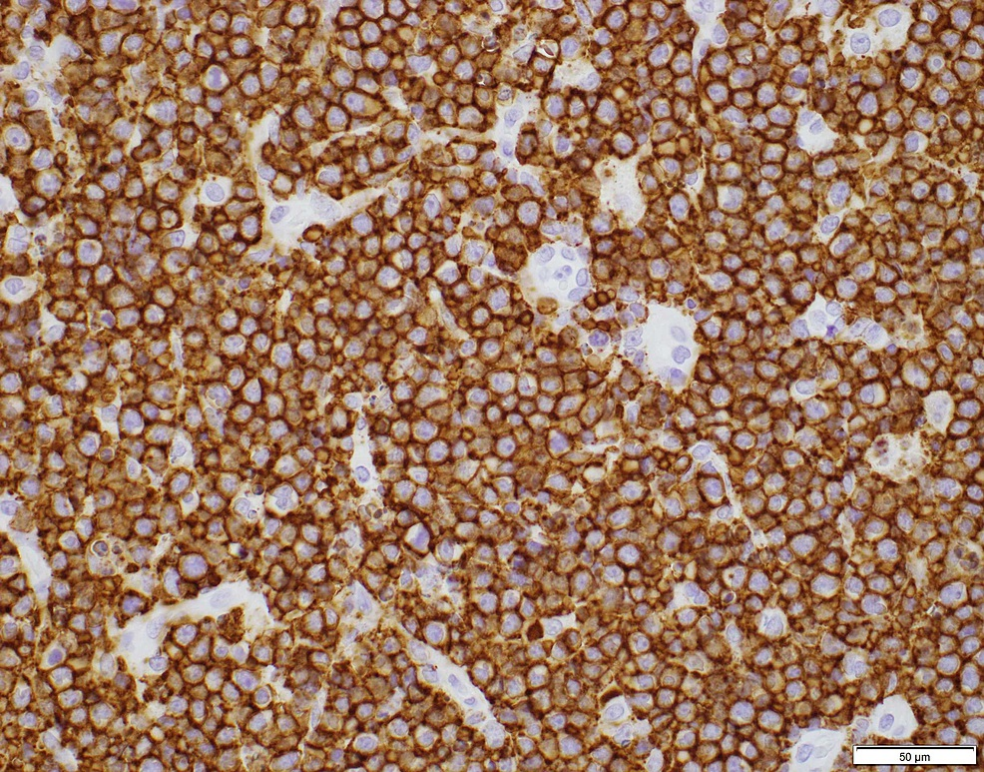
Lymphocyte infiltrate grossly positive for CD20 marker on immunohistochemical stain (40x).

**Figure 3:**
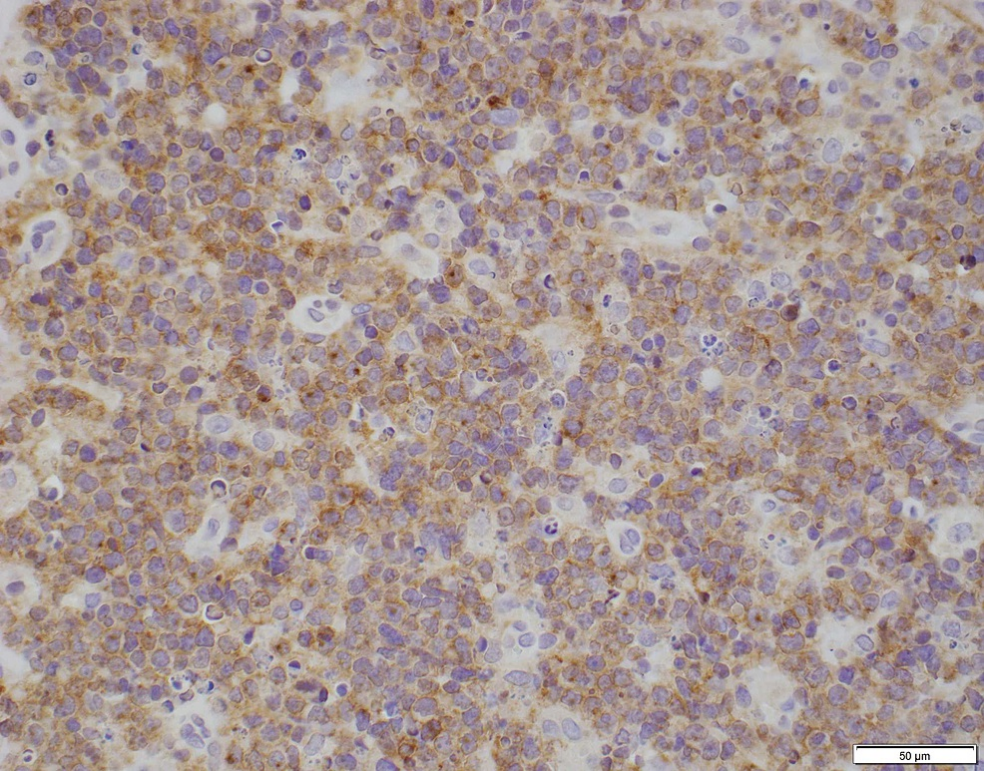
Lymphocyte infiltrate grossly positive for CD10 marker on immunohistochemical staining (40x).

**Figure 4:**
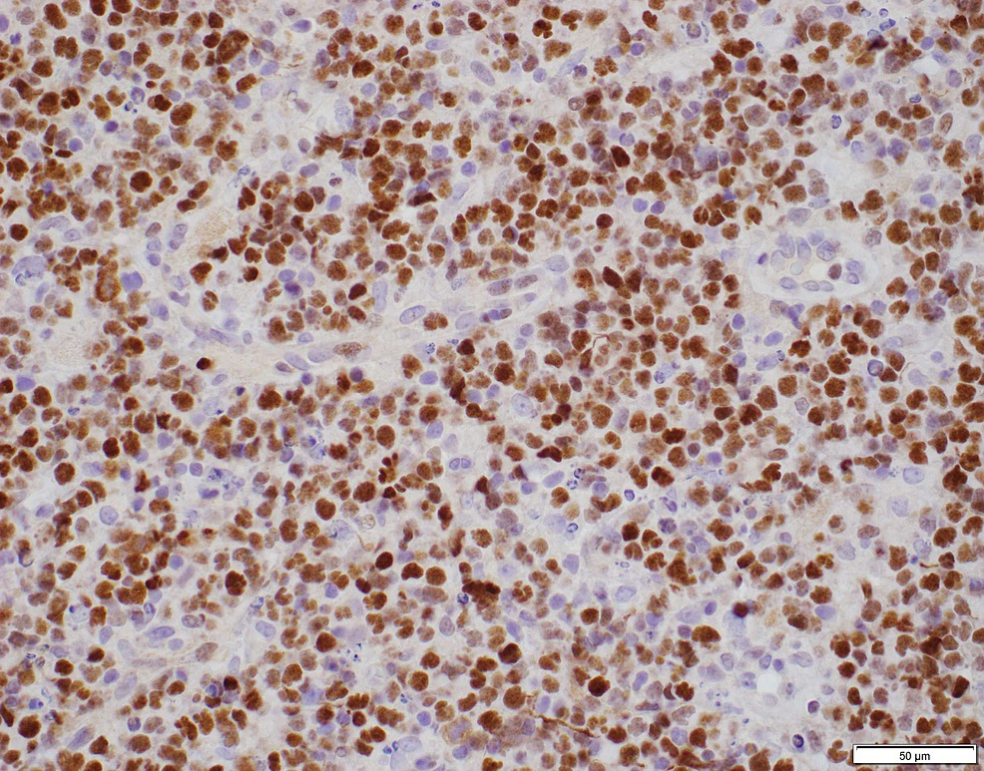
Immunohistochemical stain for Bcl-6 (40x). Positive staining of lymphoma cell nuclei.

**Figure 5:**
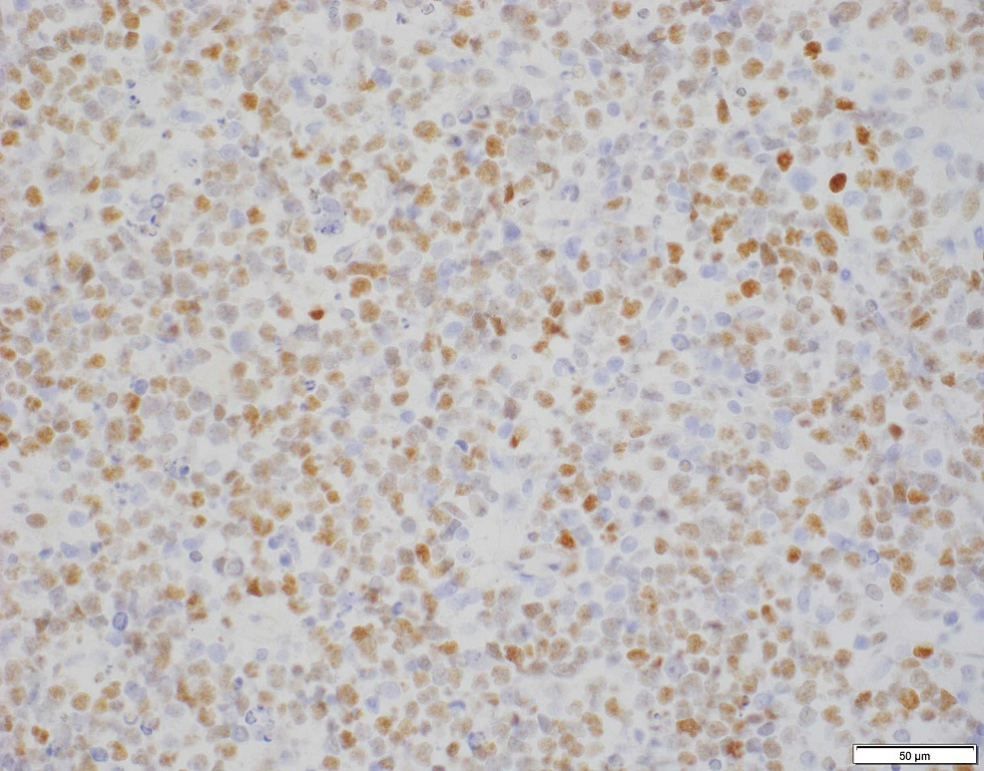
Lymphocyte infiltrate positive for c-myc on immunohistochemical staining (40x).

**Figure 6:**
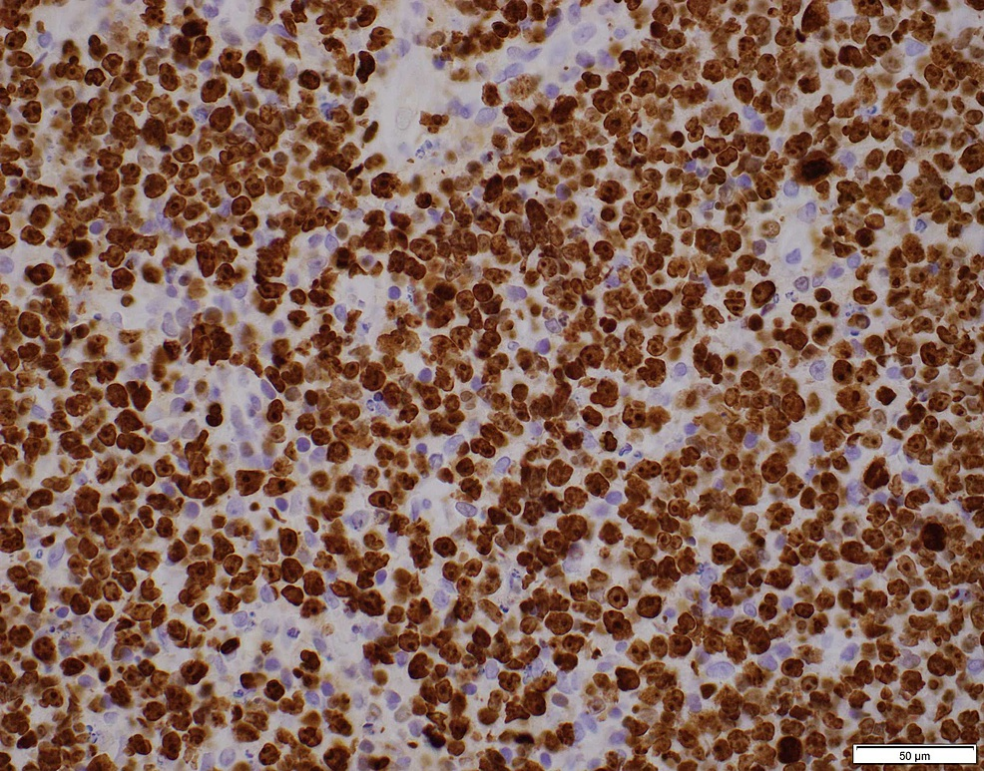
Immunohistochemical stain for Ki67, a proliferation marker (40x).

Thyroid ultrasound was performed showing gross enlargement of thyroid gland with mixed echotexture and predominant hypoechogenicity. Patient had prior thyroid ultrasounds which had no remarkable changes with recent one dated a year back from the diagnosis. Positron emission tomography/computed tomography (PET/CT) for staging showed diffusely increased metabolic activity in the thyroid gland, which was enlarged to 9.2cm x 8.2cm x 7.4cm encircling the narrowed trachea. Erosion of tracheal cartilage was also seen with thyroid tissue extending up to the superior mediastinum. There was no evidence of bone marrow, lymph node, or splenic involvement. The bone marrow biopsy was not performed. Histological examination of the thyroid specimen demonstrated large malignant lymphocytes with prominent nucleoli and vesicular nuclei replacing the thyroid parenchyma (Figure 1). Flow cytometric analysis and immunohistochemical (IHC) studies were positive for B-cell marker (CD20) and CD10, with dim to no surface immunoglobulin, thus characterizing this lymphoma as DLBCL, germinal center B-cell (GCB) subtype according to Hans classification (Figure 2, 3). Further studies demonstrated positive staining for Bcl-6 and Myc and 98% positivity for Ki67 but negative staining for Bcl-2 (Figure 4, 5, 6). Fluorescence in situ hybridization (FISH) detected MYC rearrangement but without IGH/MYC fusion. The cells were also negative for BCL6 and BCL2/IGH rearrangements. These findings ruled out the possibility of a double hit or double expressor DLBCL. 

We then started the dose-adjusted R-EPOCH regimen (rituximab, etoposide, prednisone, vincristine, cyclophosphamide, and doxorubicin). A total of eight cycles was planned with intermittent PET/CT imaging to assess treatment response. The patient’s neck swelling decreased substantially during the first cycle, and the patient was extubated without difficulty. In the meantime, after the debulking surgery, patient was continued on the same dose of levothyroxine with no change in serum TSH levels observed over the period of several months by six weekly follow-up. During the first and second cycles, the patient developed febrile neutropenia with pancytopenia, which was treated with cefepime 2g every eight hours. She was later switched to ciprofloxacin. The patient was also given intravenous pentamidine monthly, later changed to daily trimethoprim-sulfamethoxazole (TMP-SMX), acyclovir, and fluconazole for prophylaxis against pneumocystis, herpes zoster, and fungal infections. Granulocyte colony-stimulating factor (G-CSF) was also part of the regimen. Otherwise, the patient tolerated the chemoimmunotherapy regimen well and was later followed as an outpatient for the continuation of therapy. PET/CT performed after the fourth cycle of R-EPOCH (mid-treatment) showed marked resolution of hypermetabolic activity in the thyroid gland, consistent with good partial response. By the end of therapy, cycle 8, PET/CT showed no hypermetabolic activity marking first complete remission. Risks and benefits of involved site radiation therapy (ISRT) versus surveillance with quarterly PET/CT imaging review were discussed with the patient. PET/CT surveillance was chosen as the modality for further management.

## Discussion

According to the North American Association of Central Cancer Registries, non-Hodgkin lymphoma (NHL) is the seventh most commonly diagnosed malignancy in the United States. NHL mostly originates in lymph nodes and other lymphoid tissues such as Waldeyer's ring, thymus, and spleen. Meanwhile, 25%-40% of the NHLs arise in non-lymphoid tissues and are termed extranodal NHLs (EN-NHL). NHL is a heterogeneous group of lymphomas that differ in morphological, immunological, cytogenetic, and molecular characteristics. Among various subtypes of NHLs, diffuse large B-cell lymphoma (DLBCL) is the most common subtype, with an incidence rate of 30% to 40% [2]. Less than 40% of DLBCL originate in extranodal sites [3]. Primary thyroid lymphoma (PTL) accounts for only 5% of all thyroid malignancies and approximately 3% of all EN-NHL, with an annual incidence of two per one million. The most common type of PTL is DLBCL, accounting for more than 50% of the cases [4]. Recent studies have classified DLBCL into three subtypes based on gene expression profiling [5]: germinal center B-cell-like DLBCLs (GCB-DLBCL), activated B-cell-like DLBCL (ABC-DLBCL), and primary mediastinal LBCL (PMBCL).

The diagnostic evaluation of DLBCL usually begins with ultrasound as the initial imaging modality of choice. The ultrasonographic pattern of PTL shows one of three patterns: nodular, diffuse, or mixed. The appearance of heterogenous hypoechoic parenchyma with evidence of septations is primarily indicative of diffuse lymphoma [6]. Fine needle aspiration cytology (FNAC) is also an essential tool in narrowing down the differential diagnosis of thyroid diseases. However, its positive diagnostic, predictive value ranges from 25% to more than 90% [7]. Adjunctive techniques such as immunohistochemistry (IHC) and flow cytometry have been shown to enhance the accuracy of FNAC [6]. According to a study conducted by Swart et al., FNAC and flow cytometry produce sensitivity of 97% and specificity of 87% for detecting B-cell lymphoma [8]. Immunohistochemical panel stains positively for CD19, CD20, and CD45 in cases of DLBCL. For sub-classification of DLBCL, IHC panel for Bcl-2, Bcl-6, MUM1, CD10, and CD20 is adequate, where tissue samples expressing CD10 (+) or CD10 (−), Bcl-6 (+), and MUM1 (−) are considered as GCB-DLBCL; and samples expressing CD10 (−), Bcl-6 (−) or CD10 (−), Bcl-6 (+), and MUM1 (+) are considered as non-germinal center in origin [9]. An extra-aggressive subcategory of DLBCL called high-grade B-cell lymphoma is identified by the FISH analysis, which detects MYC, BCL2, and BCL-6 translocations, known as 'double-hit (DH)' or 'triple-hit (TH)' lymphoma. The DH/TH lymphoma is generally associated with poor clinical outcomes, with most patients surviving a maximum of 24 months from diagnosis. This contrasts with single MYC translocation, as seen in our patient, which shows variable clinical course [10]. 

In the past, surgical open or core biopsy was employed for definitive diagnosis of PTL. However, due to the increasing accuracy of FNAC compounded with adjunctive modalities over the years, the role of surgical biopsy is becoming limited. Nevertheless, it may still be required in cases of inconclusive FNAC results or for specialty studies such as gene rearrangements. F-18 fluorodeoxyglucose (FDG)-PET/CT is becoming increasingly frequent and recommended for staging and response assessment [7]. 

The optimal treatment for DLBCL of the thyroid is not yet established. The role of surgery remains controversial, as suggested by the study conducted at Mayo Clinic where remission rates were similar for stage I and II cases (85% and 88%, respectively) whether they received debulking surgery followed by radiation or underwent surgical biopsy only followed by radiotherapy [11]. Another study indicated a stark recurrence rate of 75% in cases of exclusive surgical treatment [12]. Nevertheless, surgery is often required for palliative purposes, especially in patients with airway obstruction who are slow responders to non-surgical regimens [7]. The modern treatment regimen has shifted gear towards the combination of chemo- and radiotherapy. The standard first-line therapy for DLBCL is a combination of rituximab with cyclophosphamide, doxorubicin, vincristine, and prednisolone (R-CHOP). The introduction of rituximab, a monoclonal antibody against CD20, present on the surface of B cells, in the chemotherapy regimen of DLBCL has brought revolutionary benefit to the patient’s outcome, especially in elderly patients [13,14]. Other recommended chemotherapeutic regimen includes DA-EPOCH-R (dose-adjusted etoposide, prednisone, vincristine, cyclophosphamide, doxorubicin, and rituximab) [3]. Studies have reported improved progression-free survival in patients with chemotherapy followed by radiotherapy. A reported recurrence rate for the primary DLBCL of the thyroid was 7.7% with combined chemoradiotherapy, 37.1% with radiotherapy alone 43% with chemotherapy alone [13]. In most instances, radiation is often introduced after three to six cycles of chemotherapy. However, some oncologists prefer the early introduction of radiation in tolerating patients [7]. Miller et al. reported significantly better progression-free survival (p=0.03) and overall survival (p=0.02) in patients treated with three cycles of CHOP followed by radiotherapy as compared to eight cycles of exclusive chemotherapy [15]. Therefore, a multimodal chemoradiotherapy regimen is considered the best treatment modality for DLBCL of the thyroid. Our patient, however, opted exclusively for chemotherapy with quarterly PET/CT surveillance as the patient had complete remission after eight cycles of DA-EPOCH-R.

## Conclusions

Primary thyroid lymphoma of diffuse large B-cell type is one of the rare hematological malignancies reported in the literature. Treatment is usually based on a case-to-case basis and oncologist's judgment. More research is needed to establish a general line of management for these malignancies to generate algorithms for treatment. We report the management with surgical debulking combined with the DA-EPOCH-R approach without using any radiation and successive imaging with PET/CT for surveillance.
